# Mechanical Dentistry

**Published:** 1854-01

**Authors:** T. L. Buckingham

**Affiliations:** Dentist.


### ARTICLE XII.

*Mechanical Dentistry.* By Prof. T. L. Buckingham, M. D., Dentist.

Our profession is divided into two grand divisions, viz. The operations on the natural teeth, and the manipulations required to replace these organs when they have been lost.

The first is termed operative dental surgery, and includes all the operations usually performed upon the natural teeth. Filling, plugging, scaling, extracting, the treatment of the diseases of the mouth, and the treatment of the irregularities of children's teeth, are all included in this branch.

To the mechanical division belong all the manipulations required to make artificial substitutes, and the apparatus necessary to correct the irregularities of natural teeth.

In the first division, nearly all of our eminent dentists are engaged, and they find sufficient to occupy all their time, without leaving them any to devote to the mechanical branch. It is, therefore, mostly performed by the younger members of the profession, or by others who have not been able to obtain a sufficient amount of operating to employ all their time. I allude now to the mechanical manipulation required to make artificial substitutes. I know that nearly every dentist has more or less artificial work done, but there are very few, except those alluded to above, who do it with their own hands.

There was a time when the workshop was indispensable to a dentist; his occupation was then considered a mechanical art, and practiced as such. His views are now changed; dentistry is no longer an art, but a profession, requiring for its operations only, a scientific knowledge of the natural teeth and their surrounding parts. I have heard many freely admit their ignorance of the mechanical branch, and almost boast that they knew nothing about it; others, although they allow that skill may be required in its operation, contend that it is but a mechanical skill, and term those who practice it, dental jewellers, or workers in *china-ware* and *pottery*.

Many now advocate the entire separation of these two branches, under the impression that both might be benefited thereby.

I propose now to examine the possibility of a separation. I admit that if they were entirely separated, it would be a decided benefit to the mechanical branch. But can they be separated? In large cities, where there is a large practice, the two branches may have offices connected, with a decided advantage to both. But where there is but a small practice, or in small towns, or in the country, I think it is impossible to separate them. No operator, in the present state of our knowledge, can be so successful as to save all the teeth that comes under his care.

There are cases presented, were so many of the teeth are diseased, and some of them so far gone, that it is necessary to sacrifice them to enable us to save the rest. These may be saved, with proper treatment and care, for a long time in a comparatively healthy condition, if nothing interferes with their natural functions. But if an artificial tooth is improperly placed by their side, or a clasp put around them, they are irretrievably lost.

Would a dentist who has a proper care for his patients, be willing to trust them in the hands of one who is admitted to be inferior to him in knowledge, skill and judgment? But we are answered, he may take the impressions, and have the work done for him. How far he can do this, and give satisfaction to his patients, we will examine in a subsequent part of this article. Our object is now to examine the possibility of a separation of the two branches. We are directed to look, as an example, at medicine, which is being divided into physic, surgery, obstetrics, &c., and with a decided advantage to each division; but this can only be done in large cities. There is, perhaps, not one surgeon practicing in the United States to every hundred physicians, and there is not one in fifty of these that practice surgery exclusively.

Even in our large cities, nearly every physician practices more or less minor surgery, and in small towns, and in the country, they are absolutely compelled to perform nearly all the surgical operations. But allowing that the dentist can send his patients to one whose ability and skill he has perfect confidence, does he by these means get rid of all the mechanical manipulations? Is he not required to be a thorough mechanic to perform his every day operations in his office? (omitting the operation of filling teeth, which, in itself, requires a great deal of mechanical skill.) The dentist has, in correcting irregularities of children's teeth, to apply every principle of mechanics he can bring to bear; the lever, fulcrum, screw, wedge, inclined plane, all have to be applied, and with all these means, and all his skill, he has sometimes the greatest difficulty to overcome the obstacles that are presented. I know of no occupation that calls so much upon the mechanical ingenuity of the operator as dentistry. He has often to shape his instruments while his patient waits in the chair, or make or alter an apparatus before he can send him away. Nearly every case presents some new obstacle, which he has to overcome by his ingenuity, requiring him not only to invent, but to construct such means as will accomplish the object desired.

In small towns, and country places, the dentist must necessarily practice both branches. In repairing, if nothing else, he cannot get his patients to wait until he sends his work away to be done. What would they think of a dentist who would ask them to wait three or four days, while he sent the case away to have a tooth or a clasp soldered on, which should take only about half an hour to do it. We are compelled to do these repairs while the patient waits in the parlor, and we pity the dentist who is not able to do them himself. We have known dentists to leave their patients sitting in their chairs while they walked a mile to have a case repaired, when it would not take half as long to repair it, as it did to walk the distance.

We might relate cases to show the importance of a dentist being able to do his own work, but it is not necessary. We all can imagine the mortification that must be felt by a person who has been wearing a set of artificial teeth, when they are compelled to go into company without them. Ladies generally shut themselves up in their rooms, and deny all company; and gentlemen, when they are compelled to attend to their business, do it with the greatest reluctance. One remarked to us the other day that he would almost as leave be seen without his nose as without his teeth.

Admitting that there is a mechanical dentist near at hand, to whom the operator can send his patients to have artificial work done, is it policy for him to do so? Patients, generally, have not the ability to discriminate between the capacity of one who is capable of making a piece of mechanism to be used in the mouth, and one who does what they consider a mechanical operation in it. We have known dentists whose practice has been built up or materially benefited by operators recommending patients to them, to have artificial work done.

Let us examine, and see whether a dentist can do justice to his patients by merely taking the impressions, and having the work made for him. Our object, when we insert artificial teeth, should be to imitate the lost organs as nearly as possible, or at least to make such substitutes as will suit the case. If we will examine all the sets of natural teeth that come under our notice, we will find that no two are exactly alike, and yet many of them are very handsome, far beyond any artificial substitute we can expect to make; and what generally constitutes their beauty, is the harmony with which they agree with the other features of the face.

Teeth which would be handsome in one mouth would be decidedly ugly in another; a person with a full, round face, may have short, thick teeth, worn off half way to the gum, and yet they may give a better expression to the mouth than other shaped teeth would. Another tall, thin person, has long, narrow teeth, the upper row lapping the under very much. These are just the teeth to give a pleasing expression to his face. Between these two extremes we have every variety; some with jaws so narrow and contracted, that their four natural incisors scarcely occupy space enough for two; others, with mouths so large that we can hardly find teeth wide enough to fill the space.

Again, we have patients of all ages, requiring artificial teeth; some of sixteen, others of sixty years of age. Natural teeth show the effect of time and wear upon them as much as any of the other organs of the body. Even in animals, whose teeth are formed much more regularly and uniformly than the human teeth. They are a criterion by which we judge their age, and we can tell, after they have been extracted from the jaw, whether they belong to the old or young animal.

I think I have shown—if it was necessary to show what every body knows—that all sets of natural teeth are not alike. I might now proceed to show that all sets of artificial teeth are made as much alike as the cases will permit.

To be sure, they are in some cases made a little larger than they are in others, (but this is only because the jaws are larger,) and there is, perhaps, two or three shades of color.

With these exceptions, they are made as near alike as the mechanical dentist can make them. In fact, he forms in his mind, or makes himself a model of the most perfect shaped teeth he can conceive of. This he makes his model to go by in all cases. It makes no difference whether the teeth are for an old person or a young one, they are all made alike.

This must necessarily be the case, when the person who makes the teeth has never seen the patient. How can he form them to suit features that he has never seen? If we were to send to an artist who makes artificial limbs, and request him to make a leg or an arm to supply one that had been lost, and send him nothing to go by but the impression of the stump and the length it was to be, would any body suppose that he could make one that would correspond with the natural limb? or, if a person should require an artificial nose, and was merely to send the impression of the surface it was to cover, could any body expect that it would correspond with the other features of the face?

These are as explicit directions as are generally sent to have artificial teeth made. A piece of wax, adjusted upon a plate that fits the jaw, and a tooth the color they are to be, is all the direction usually considered necessary. Then should we be surprised that we find artificial teeth all made alike; or when we find a stout, athletic man, whose features show that he has been exposed to all kinds of weather and has followed some laborious occupation all his life, having the same shape and style of artificial teeth that are placed in a delicate lady's mouth?

Even where the dentist has the laboratory in the house with his office, and has an assistant to do the work, his presence is almost constantly required if he wishes to give satisfaction to his patients, and many of the manipulations he must positively perform with his own hands. He cannot convey the idea of the expression of the patient's face by the most explicit descriptions he can give, and without this it is impossible to arrange teeth to harmonize.

Let us point out some of the manipulations required to insert artificial teeth, and we will see how much can be delcgated to another. After the impression has been taken and the plaster cast made, it is necessary (when there are teeth on it) to trim it more or less. It is impossible to get a perfect impression of natural teeth with any substance we now use. Teeth are larger at their crowns than they are at their necks, and generally their points approximate where teeth between them have been lost. This will cause the wax to be dragged up, so that the impression will be imperfect, and the cast require trimming, which should be done by one who has seen the mouth. The metal casts can now be made, a plate struck up, and the clasps adjusted, by an assistant. They should now be tried in the mouth, each part separately, and adjusted to fit the parts properly.

They should then be cemented together and tried in the mouth, and arranged so as to fit without forcing or dragging the natural teeth in any direction—all this requires the presence of the operator. The different parts of the apparatus can now be soldered together by any person capable of doing it.

The teeth are now to be selected and arranged. This is the most important part of the whole operation. This cannot be delegated to another, but must be performed by the dentist himself, if he wishes to give satisfaction to his patients. When the teeth have been tried in the mouth and arranged to suit, the case may be finished by any person competent to do it.

If we now divide the time required to perform these manipulations as we have separated them, we will find that the dentist will have to be engaged at least twice as long as his assistant; in fact, there is little of the whole operation that can be delegated to another.

If we have succeeded in showing that there should be some harmony between the teeth and the other organs of the face, we think we have demonstrated fully the necessity of the person who takes the impression and puts the case in the mouth, doing at least two-thirds of the work with his own hands.

We have said nothing in this article about the utility of artificial teeth, or the benefits the wearer may derive from them beyond their mere appearance. Their importance in mastication, and the effect they have upon the voice, may form a subject for another article—*Dental News Letter.*

